# Editorial: AI-enabled breakthroughs in computational imaging and computer vision

**DOI:** 10.3389/frai.2026.1811469

**Published:** 2026-04-07

**Authors:** Liping Zhang, Xiaobo Li

**Affiliations:** 1 Athinoula A. Martinos Center for Biomedical Imaging, Harvard Medical School, Boston, MA, United States; 2 Department of Radiology, Massachusetts General Hospital, Boston, MA, United States; 3 Laboratory of Biomedical Imaging and Signal Processing, Department of Electrical and Electronic Engineering, The University of Hong Kong, Pokfulam, Hong Kong SAR, China; 4 School of Marine Science and Technology, Tianjin University, Tianjin, China; 5 Key Laboratory of Mariculture (Ministry of Education), Fisheries College, Ocean University of China, Qingdao, China

**Keywords:** artificial intelligence, complex environments, computational imaging, computer vision, medical imaging, optical systems, pattern recognition

Artificial intelligence (AI), particularly modern pattern-recognition methods, is transforming how visual information is acquired, reconstructed, interpreted, and translated into decisions. In computational imaging and computer vision, progress is no longer defined by model accuracy alone. It increasingly depends on whether methods remain reliable under practical constraints, including limited annotations, heterogeneous acquisition conditions, domain shift, and runtime requirements. Recent advances in medical image segmentation and accelerated reconstruction (Zhang et al., 2021; Zhang and Chen, 2023; Zhang et al., 2024), together with progress in cloud detection for remote sensing (Yang et al., 2019) and imaging through scattering media and underwater degradation (Li et al., 2022; Zhou et al., 2023; Li et al., 2025), reflect this broader shift toward robust, deployment-aware AI.

This Research Topic was organized around this translational agenda. Its purpose was to showcase work at the intersection of AI and imaging technologies that is both methodologically innovative and practically meaningful, spanning medical imaging, optical systems, and complex real-world vision settings. Although the four accepted contributions address different application domains, they converge on a common principle: durable progress comes from combining strong representation learning with task-aware pipeline design and realistic evaluation.

Ren et al. address a persistent challenge in medical AI: retaining the representational strengths of large pre-trained vision models while preserving computational feasibility. Their distillation framework transfers knowledge from a large teacher model to a lightweight student model for medical image diagnosis. From a practical perspective, this study shows that foundation-model priors can be leveraged without sacrificing deployability, which is critical for clinical integration.

Habe et al. investigate real-time abnormality detection in wireless capsule endoscopy. In this setting, clinical utility depends on throughput as much as precision. By adapting transformer-based RT-DETR models and analyzing scale-dependent trade-offs, the study frames performance as an operating-point decision rather than a single benchmark score. This framing is directly relevant to clinical deployment, where latency, reliability, and detection quality must be balanced according to workflow requirements.

Ramya and Minu take a coordinated multi-stage approach to oral squamous cell carcinoma grading, combining enhancement, augmentation, segmentation, and transformer-assisted classification. The contribution is as much methodological as it is architectural. Their study shows that integrating multiple components within a structured pipeline can improve the robustness of pathology tasks where data are limited and class structure is challenging.

Shah et al. extend the collection beyond hospital-centered imaging into agricultural vision. Their DeepSVM framework for the early diagnosis of dome galls in *Cordia dichotoma* combines deep feature learning with margin-based classification. The study highlights the ongoing importance of hybrid deep–classical designs in field conditions, where visual variability is high, and data curation is less controlled. This contribution closely aligns with the Research Topic's emphasis on AI for complex, real-world environments.

Taken together, the collection points to three converging directions for the field. First, efficiency-aware model design is now foundational: model capacity must be matched to practical computing constraints. Second, pipeline-level integration is often decisive in constrained data regimes, where preprocessing, localization, and classification are tightly coupled. Third, evaluation under operational conditions–including latency, robustness, and generalization beyond curated benchmarks–should be treated as a primary scientific criterion rather than a downstream engineering add-on.

In summary, this Research Topic reflects a maturing stage of AI research in imaging, vision, and pattern recognition. The field is moving beyond isolated model improvements toward integrated systems that are accurate, efficient, and reliable in the face of practical uncertainty. We hope that this collection encourages closer collaboration among AI researchers, imaging scientists, and domain experts and supports continued innovation at the intersection of learning algorithms, imaging physics, and deployment-oriented system design.
